# Cholinergic innervation and ganglion cell distribution in Hirschsprung’s disease

**DOI:** 10.1186/s12887-020-02299-z

**Published:** 2020-08-24

**Authors:** Anne K. Braczynski, Stefan Gfroerer, Rudi Beschorner, Patrick N. Harter, Peter Baumgarten, Udo Rolle, Michel Mittelbronn

**Affiliations:** 1 Department of Neurology, RWTH Aachen University Hospital, Aachen, Germany; 2 Department of Physical Biology, Heinrich-Heine University, Düsseldorf, Germany; 3 Institute of Biological Information Processing (IBI-7: Structural Biochemistry, Forschungszentrum Jülich, Jülich, Germany; 4 Institute of Neurology (Edinger Institute), Goethe University, Frankfurt, Germany; 5 Department of Pediatric Surgery, Helios Hospital Berlin-Buch, Berlin, Germany; 6 Institute of Pathology and Neuropathology, Eberhard-Karls University, Tuebingen, Germany; 7 German Cancer Consortium (DKTK), Heidelberg, Germany; 8 German Cancer Research Center (DKFZ), Heidelberg, Germany; 9 Frankfurt Cancer Institute (FCI), Frankfurt am Main, Germany; 10 Department of Neurosurgery, Goethe University, Frankfurt, Germany; 11 Department of Pediatric Surgery, University of Frankfurt am Main, Frankfurt, Germany; 12 University Children’s Hospital, Goethe University, Frankfurt, Germany; 13 Department of Oncology (DONC), Luxembourg Institute of Health (LIH), Strassen, Luxembourg; 14 Luxembourg Centre for Systems Biomedicine (LCSB), University of Luxembourg, Luxembourg City, Luxembourg; 15 National Center of Pathology (NCP), Laboratoire national de santé (LNS), 1, Rue Louis Rech, L-3555 Dudelange, Luxembourg; 16 Luxembourg Center of Neuropathology (LCNP), 1, Rue Louis Rech, L-3555 Dudelange, Luxembourg

**Keywords:** Hirschsprung’s disease, Colon, Recto-sigmoid, AChE, Aganglionosis

## Abstract

**Background:**

The diagnostic gold standard of Hirschsprung’s disease (HD) is based on the histopathological assessment of colorectal biopsies. Although data on cholinergic innervation and ganglion cell (GC) distribution exist, only few studies have examined these two key features together. We assessed the pattern of cholinergic innervation and the amount of GCs in colorectal specimens of 14 HD patients.

**Methods:**

We established a semi-quantitative score for cholinergic innervation using acetylcholinesterase (AChE) enzyme histochemistry and quantitatively analyzed the number of GCs via NADH tetrazolium reductase (NADH) enzyme histochemistry. We examined both the entire length of the resected specimens as well as defined areas of the transition zone of both pathological and healthy appearing segment.

**Results:**

High AChE score values were associated with absence of GCs, and AChE scores were inversely correlated with the number of GCs. Nevertheless, we observed several cases in which one of the two features revealed a normal distribution pattern, whereas the other still displayed pathological features.

**Conclusions:**

Our data support the need for transmural colon biopsies, to enable the best evaluation of both cholinergic innervation and GCs for a reliable assessment of HD.

## Background

Hirschsprung’s disease (HD) is a rare cause of constipation in infants that is microscopically characterized by congenital absence of GCs in a variable extension mainly affecting the rectum and sigmoid colon. HD has a prevalence of approximately 1:5000 in newborns. Male babies are more frequently affected, with a ratio of 4:1 (male:female) [1]. A register-based study has described a slight increase in incidence over the recent years [2]. The clinical symptoms of HD comprise severe chronic constipation, ongoing defecation problems and occasionally bowel obstruction. Most newborns with HD present with a delayed meconium passage [3]. In infancy, a postnatal passage time of longer than 24–48 h combined with defecation problems is clinically suggestive for HD [4]. The treatment of choice for HD is the surgical resection of the affected aganglionic segment. The transition zone, which is only partially populated by GCs, should also be resected. The operation technique should preserve the anorectal function, which is usually achieved by connecting the distal functional bowel with the sphincter muscle at the area of the dentate line [5]. Despite proper surgical treatments, the quality of life may remain negatively affected during childhood [6]. Most cases of HD occur sporadically, nevertheless, genetic causes of this disorder have been identified [7, 8]. The most frequent mutations affect the *RET* gene that codes for a receptor tyrosine kinase [9]. Approximately 50 different *RET* mutations have been reported in HD patients, accounting for 50% of familial cases of HD and 15 to 20% of sporadic cases of HD [7].

The diagnosis of HD is histopathologically confirmed by the absence of GCs in the enteric plexus of the distal rectum (usually several biopsies ranging between 1 and 6 cm above the dentate line [10–12] via either superficial suction [13] or a full-thickness (transmural) specimen [14]). Intraoperative diagnostics to detect GCs is most frequently based on the hematoxylin and eosin (H&E) staining of cryosections. This method can be combined with enzyme histochemistry for (i) the assessment of cholinergic nerve fibers using acetylcholinesterase (AChE) [15] and (ii) the detection of ganglion cells via lactate dehydrogenase (LDH), succinate dehydrogenase (SDH), and NADH tetrazolium reductase reactions (NADH) [16, 17]. Although average values exist concerning the number and distribution of ganglion cells in normal intestines and intestines affected by HD [18], these values are highly dependent on the staining method [14]. Histopathologically, the diagnosis consists of an absence of GCs and the presence of hypertrophic AChE-positive nerve fibers, however little is known about the correlation of the GC distribution pattern and the cholinergic innervation HD cases. Therefore, in the current study, we aimed at analyzing both the extent and distribution pattern of cholinergic innervation in the mucosal layer in relation to the density of GCs in the myenteric plexus in a cohort of 14 HD patients.

## Methods

### Patient data

HD patients who were included in this retrospective, monocentric study underwent transanal pull through (TAPT) surgeries at the Department of Pediatric Surgery, University Hospital Frankfurt/Main, Germany. In all cases, the diagnosis of HD was confirmed by transanal rectal biopsies prior to TAPT surgeries. The specimens (for detailed information please see Table 1). were independently reviewed by at least two experienced neuropathologists (AKB, PNH, MM) from the Institute of Neurology, according to the diagnostic criteria that were proposed by Knowles [19]. Patients with long-segment diseases were excluded from this study. The ages at the time of the surgeries ranged from 28 days to 25 months (mean age: 6.5 months). The study cohort consisted of 11 male (m) and 3 female (f) patients.

**Table 1:** Sample data

ID	length of aganglionic segment/ total length (cm)	swiss roll	TZ 0 cm	TZ 5 cm	TZ 10 cm	TZ 15 cm
**1**	7/17	X	X	X	X	
**2**	6/16	X	X	X	X	
**3**	11/25	X	X	X	X	
**4**	5/17	X	X	X	X	
**5**	4/12	X	X	X		
**6**	13/30	X	X	X	X	X
**7**	15/32	X	X	X	X	X
**8**	13/25	X	X	X		
**9**	9/19.5	X		X	X	
**10**	12/17	X				
**11**	7/25	X				
**12**	28/43	X				
**13**	n.a./12.5	X				
**14**	16/29	X	X	X	X	

Abbreviations: *X* specimen available, *n.a.* not available

### Ethical statement

The parents of the patients gave written informed consent for the surgery. The use of patient material was approved by the ethical committee of the Goethe University Frankfurt, Germany (GN 438/19).

### Sampling

**Fig. 1:**
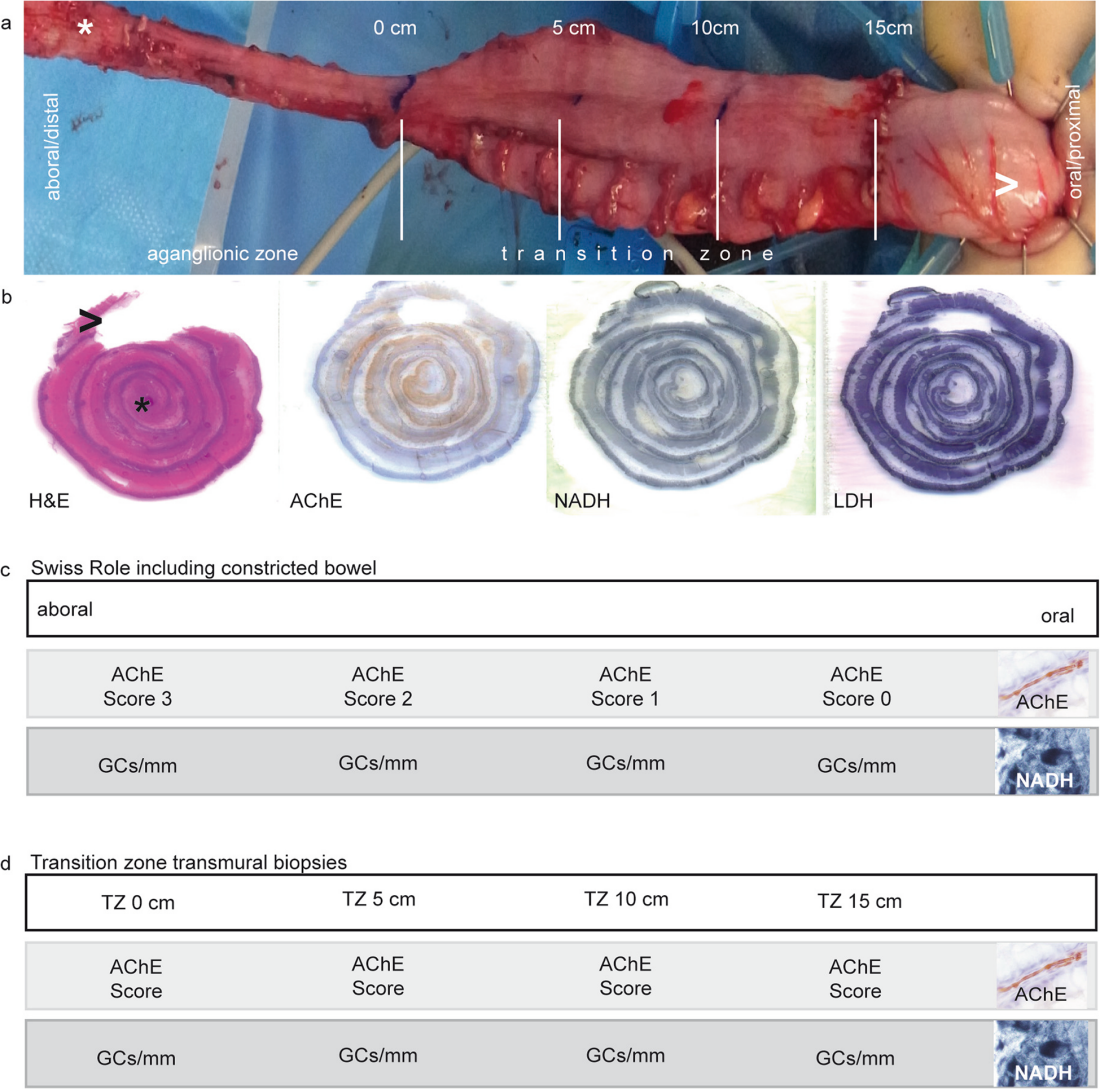
Sampling and study design. **a** Intraoperative aspect of rectosigmoid colon during TAPT procedure. There was a notable change of diameter at the beginning of TZ (TZ 0 cm). White lines mark the biopsy sites at 5, 10 and 15 cm proximal to the beginning of the transition zone (TZ 0 cm). (Asterisk indicating aboral/distal end of colon, arrowhead the oral/proximal resection region). **b** Swiss roll specimens stained for H&E, AChE, NADH and LDH (asterisk and arrowhead as described in (a)). **c** First, we analyzed the swiss rolls of the full-length specimens and located areas with each of the four grades of the AChE-Score. In the same region, we counted GCs. **d** Second, we analyzed the specimen TZ 0, 5, 10 and 15 cm for their respective AChE-Score (AChE) and counted the GC/mm in the corresponding area (NADH)

Partial colectomy specimens were examined and measured (*n* = 14). Additionally, each resected bowel segment was macroscopically analyzed with regard to the changes in diameter in the zones of bowel obstruction. The length between the proximal resection margin and the beginning of the dilated colon was documented **(**Fig. 1a**)**. Swiss roll specimens were prepared according to the following guidelines. First, from each of the entire rectosigmoidal specimens, a longitudinal, 0.5–1 cm wide strip was excised in paramedian orientation to the antimesocolic tenia. Afterwards, the strip of the intestinal wall was coiled in caudocranial orientation [20] in cryogel (Tissue Tek O.C.T., Sakura, Alphen aan den Rijn, Netherlands) attached to a cork plate and snap frozen (see below). Swiss rolls were then used to analyze the overall innervation pattern. The “serial biopsies” (referring to the specimens from the transition zone (TZ): TZ 0 cm, 5 cm, 10 cm and 15 cm) were prepared as follows: the beginning of the change in the diameter was defined as the beginning of the transition zone and referred to as TZ 0 cm. A horizontal incisional antimesocolic biopsy of approximately 3 cm × 1 cm was excised at 0 cm. Further rostral biopsies were performed at 5 cm, 10 cm and 15 cm (TZ 5 cm, TZ 10 cm, TZ 15 cm) **(**Fig. 1a**)**. The biopsies were fixed in cryogel on the cryostat specimen holder. All specimens were frozen at − 80 °C in isopentane, which was precooled in liquid nitrogen. Then, 15-μm-thick sections were cut with a manual cryostat system (Leica CM 1900, Wetzlar, Germany).

### Staining and enzyme histochemistry

Cryosections were stained with H&E, NADH reaction, LDH reaction and AChE reaction, according to standard protocols **(**Fig. 1b**)**. The incubations were performed at 37 °C for 120 min (NADH), 45 min (LDH) and 90 min (AChE). Stained sections were mounted in Entellan (Merck Millipore, Darmstadt, Germany).

### Scoring

**Fig. 2:**
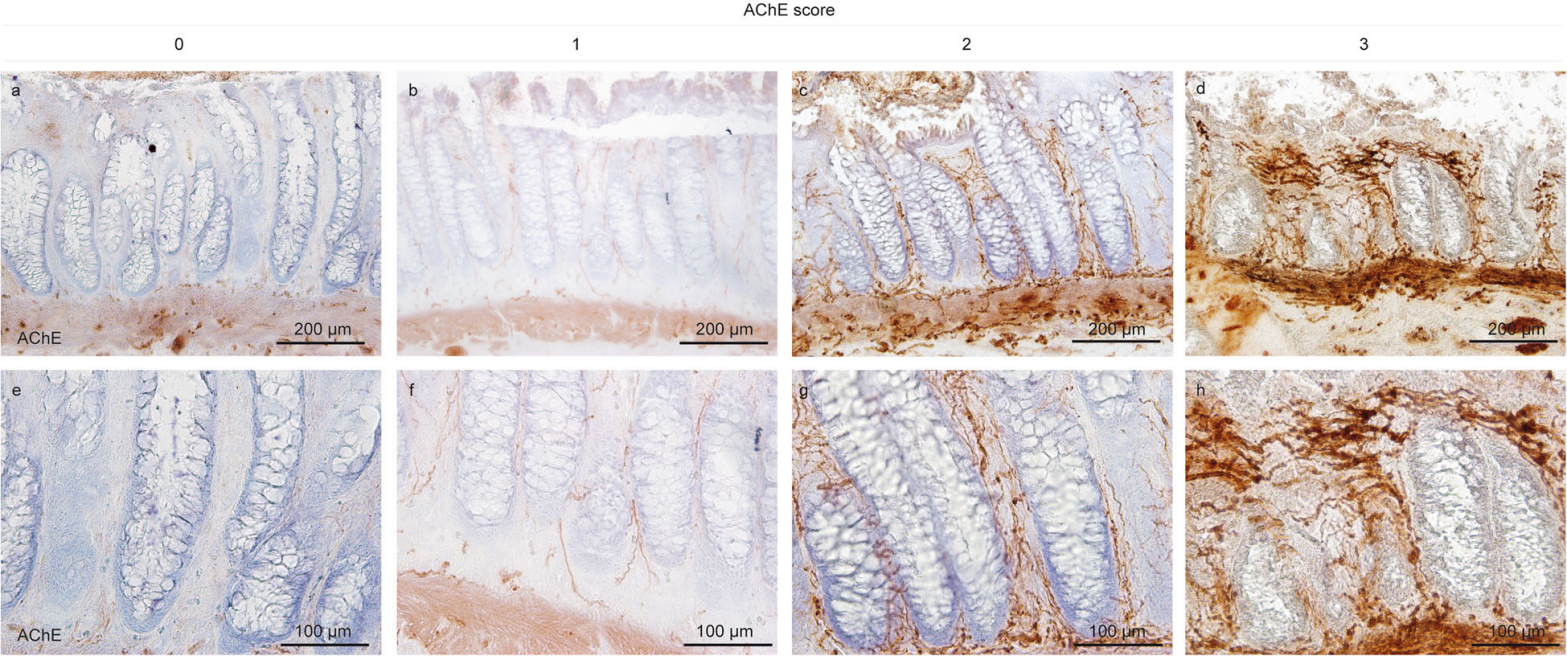
AChE scores of cholinergic innervation in the lamina propria mucosae. Representative areas of the mucosal layer with cholinergic innervation. Score 0: absence of AChE-positive fibers (**a**, **e**); score 1: mild cholinergic innervation (**b**, **f**), score 2: moderate cholinergic innervation (c, g); and score 3: severe cholinergic innervation (**d**, **h**). Scale bar a-d 200 μm; e-h 100 μm

**Fig. 3:**
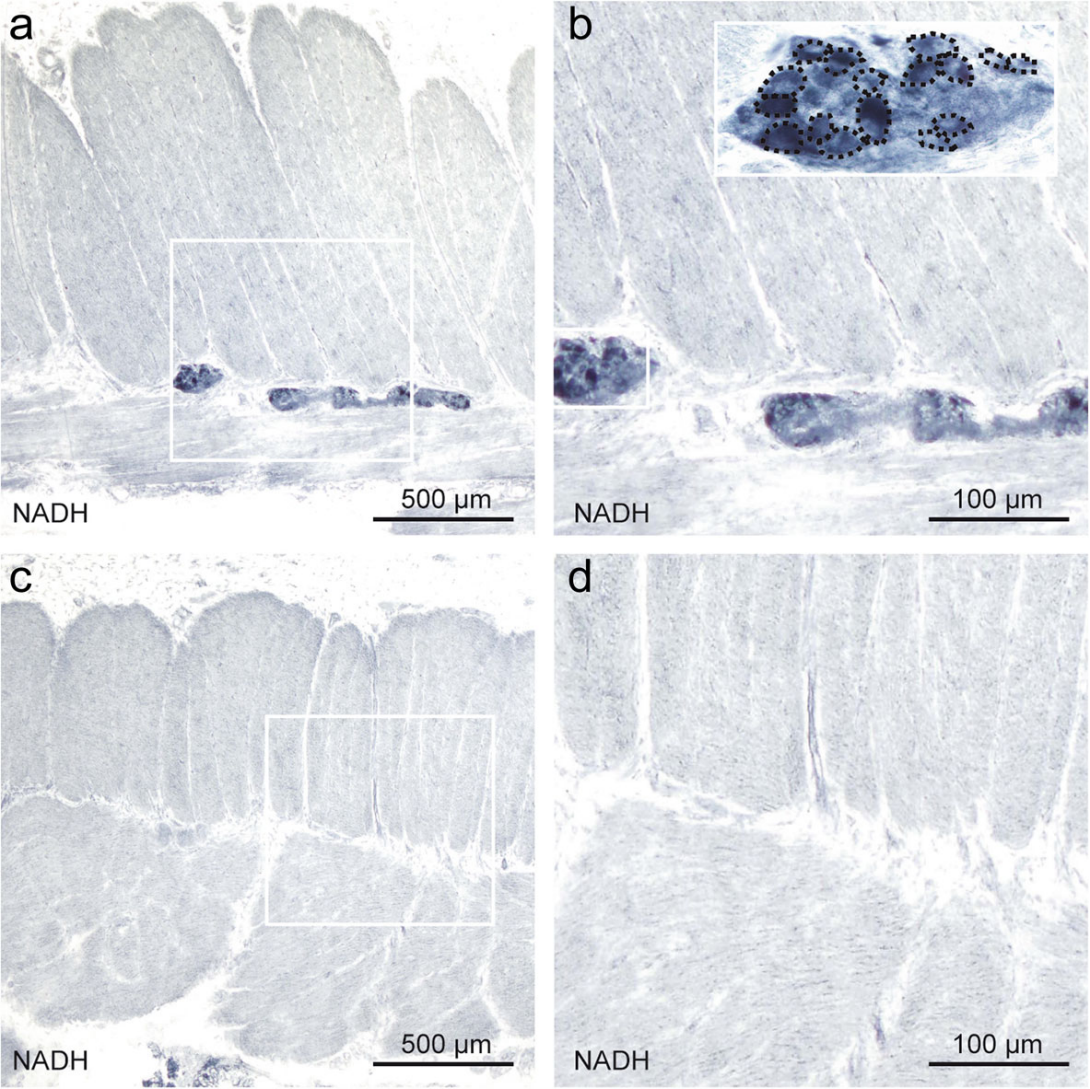
Ganglion cell count in the myenteric plexus. **a** and **b** Healthy bowel areas with normal GC density. Dashed lines indicating GCs. **c** and **d** Classic HD pathology with complete absence of GC. NADH. Scale bar **a**, **c**: 500 μm; **b**, **d**: 100 μm

Before performing semi-quantitative and quantitative analyses, a joint training was done to reduce inter-observer variability (AKB, PNH, MM). In case of doubt during quantification, a joint analysis was performed until agreement was reached. A semi-quantitative scoring system was applied to evaluate the pathological cholinergic innervation in the mucosal layer **(**Fig. 1c, d**)**. The four grades of the AChE score were defined as follows: score 0, no AChE-positive nerve fibers in the mucosal layer **(**Fig. 2a, e**)**; score 1, mild positivity (1 fiber per crypt within the lamina propria mucosae (LP)) **(**Fig. 2b, f**)**; score 2, moderate positivity (up to 2 fibers per crypt) **(**Fig. 2c, g**)** and score 3, strong positivity with 3 or more fibers per crypt **(**Fig. 2d, h**)** in the LP. GCs were counted within the myenteric plexus using NADH enzyme histochemistry **(**Fig. 3**).**

Based on the AChE scoring, two different analyses were performed. First, we determined the number of GCs/mm in the NADH stainings in areas corresponding to each distinct AChE score in the swiss roll **(**Fig. 1c**.** For raw values see Supplementary Table 1**)**. In most specimens, areas meeting each grade of the AChE score were present. For the second analysis, transition zone samples were analyzed for AChE scores, as well as for the number of GCs/mm in the corresponding area **(**Fig. 1d**.** For raw values see Supplementary Table 2**)**. The length of the myenteric plexus segment was determined by an open polygon line using image analysis software analySIS docu (Olympus, Hamburg, Germany). Depending on the quality of the specimens, the amount GCs in a segment of 20–40 mm were counted. Absolute GC count was then normalized to the length of the respective myenteric plexus segment to compare GC density in GC/mm. The imaging and evaluation were performed using a light microscope (Olympus BX41) at a magnification of 40x (length) or 400x (cell count).

### Statistical analysis

**Fig. 4:**
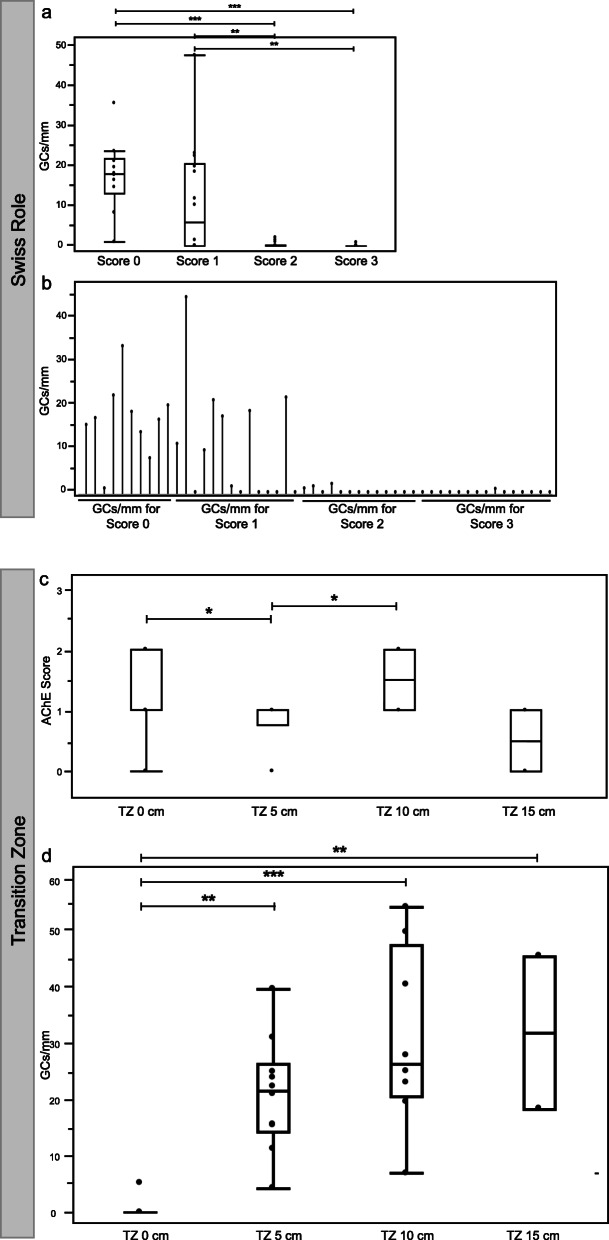
Analysis of the Swiss Role and AChE score and GC distribution in HD transition zone. **a** GCs/mm in areas assigned to AChE score 0 to 3 in the full length swiss roll preparations. **b** Single case overlay diagram. GCs/mm per AChE score are grouped according to AChE-Score 0 to 3 in a. In 4 cases no areas with score 0 were detectable. **c** Cholinergic innervation as measured by the AChE Score and **d** GCs/mm in the TZ samples (TZ 0 cm, 5, 10 and 15 cm) (*: *p* < 0.05; **: *p* < 0.01; ***: *p* < 0.001)

For statistical analyses, we used single case overlay diagrams (Fig. 4b). Significance levels were calculated by using an ANOVA and Tukey’s test (Fig. 4a, c, d**)**. A significance level of α = 0.05 was determined for all tests. Statistical analyses were performed by using the JMP 11.0 software (SAS, Cary, NC, USA).

## Results

### AChE score negatively correlated with ganglion cell count

**Fig. 5:**
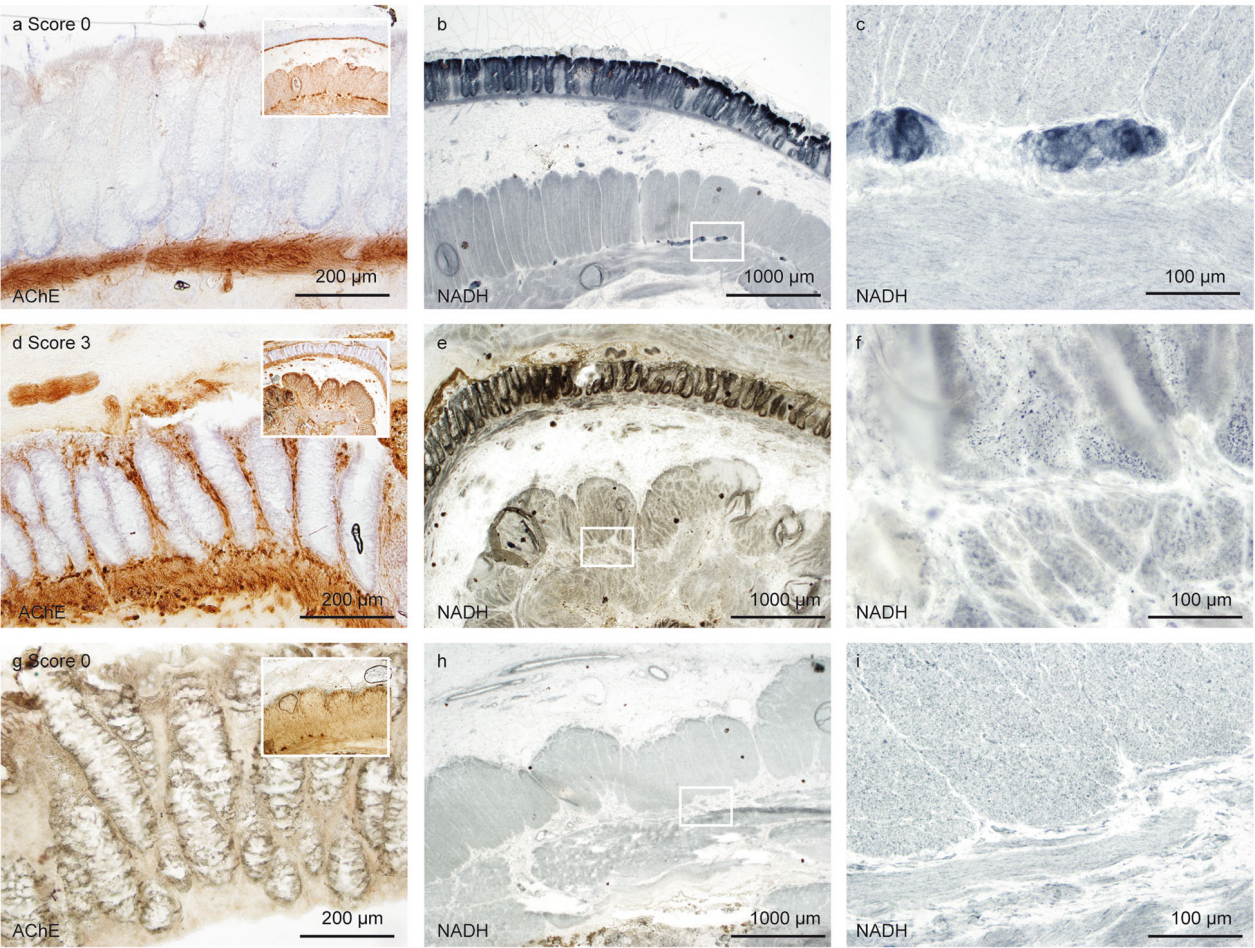
Discrepancy of ganglion cell count in areas with normal appearing AChE distribution. In general, an AChE score 0 (**a**) corresponded to GC population counts as described in [17] (**b** and **c**), while an AChE score 3 (**d**) corresponded to the absence of GCs (**e** and **f**). However, case ID 5 showed no cholinergic innervation (AChE score 0, **g**) associated with absence of GCs (**h** and **i**). Scale bar a, d, g 200 μm; b, e, h 1000 μm; c, f, i 100 μm

In areas with no AChE-positive nerve fibers in the mucosal layer (score 0; Fig. 5a-c), a median of 17.8 GCs/mm (min.: 0.9; max.: 35.4) was observed. In areas with mild AChE positivity (score 1), a median of 5.8 GCs/mm was counted (min.: 0; max.: 47.2). In areas with moderate AChE positivity (score 2), a median of 0 GCs/mm (min.: 0; max.: 2.0) was counted. In areas with severe AChE positivity (score 3; Fig. 5d-f), a median of 0 GCs/mm (min.: 0; max.: 0.8) was observed. There were significant differences in the GC counts when comparing scores 0 vs. 2 and 0 vs. 3 (each *p* < 0.001) as well as scores 1 vs. 2 and 1 vs. 3 (each *p* < 0.01) (Fig. 4a).

### Low AChE score values were associated with a high number of ganglion cells, but the absence of pathological cholinergic innervation was not a reliable indicator for a regular ganglion cell count

**Fig. 6:**
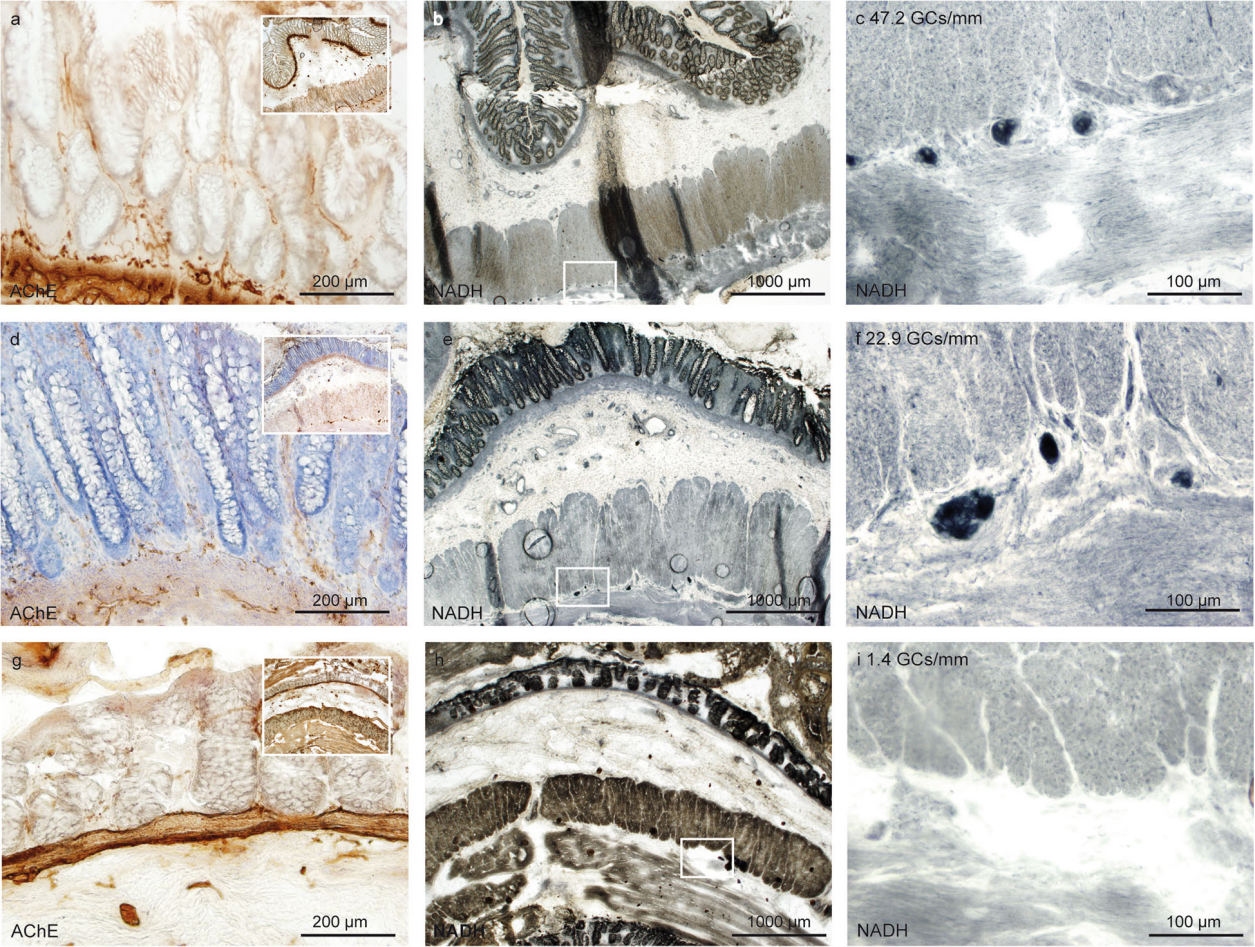
Variation of ganglion cell distribution in cases with similarly pathological AChE reaction. Three cases with AChE Score 1 are shown (**a**-**c**: Case ID 2, **d**-**f**, Case ID 13, **g**-**i**, Case ID 7). While the amount of pathologic cholinergic innervation is comparable, GC counts per mm varies from 1.4/mm (**h**-**i**, Case ID 7) to 47.2/mm (**b**-**c**, Case ID 2). Scale bar a, d, g 200 μm; b, e, h 1000 μm; c, f, i 100 μm

Moderate or severe pathological cholinergic innervation in the LP was associated with complete absence of ganglion cells in the myenteric plexus **(**Fig. 4b and Fig. 5g-i**)**. However, the absence of or presence of only very few AChE positive fibers in the mucosa did not assure a regular GC distribution in all cases. In 4 out of a total of 14 resection specimens, there were no areas detected that could be assigned to an AChE score “0”. Furthermore, there were two cases with clear aganglionosis at the aboral resection margin, however showing no sign of pathological cholinergic innervation (AChE score 0) in the respective segment (exemplarily Case ID 5, Fig. 5g-i). The colonic areas with mild cholinergic innervation corresponding to an AChE score of “1” still exhibited a considerable variation in the GC counts, with variations between 1.4 GCs/mm (Case ID 7) and 47.2 GCs/mm (Case ID 2) **(**Fig. 6**)**.

### AChE scores did not continuously decrease in the transition zone

Serial biopsies from the defined areas within the transition zone were collected **(**Fig. 1a and d**)**. Depending on the available material and the lengths of the resected specimens, the sample counts varied (TZ 0 cm: *n* = 10; TZ 5 cm: *n* = 11; TZ 10 cm: *n* = 9 and TZ 15 cm: *n* = 2). The evaluation of the pathological cholinergic innervation in the transition zone specimens revealed a median AChE score of 2 at TZ 0 cm (min.: 0; max.: 2), a median score of 1 at TZ 5 cm (min.: 0; max.: 1), a median score of 1.5 at TZ 10 cm (min.: 1; max.: 2) and a median score of 0.5 at TZ 15 cm (min.: 0; max.: 1). The decrease between TZ 0 cm and TZ 5 cm and the increase between TZ 5 cm and TZ 10 cm were both significant (*p* < 0.05) **(**Fig. 4c**)**.

### Ganglion cell counts increased in the proximal region of the transition zone and remained stable rostrally

The GCs/mm were counted in the transition zone biopsies. At the most distal point of the transition zone (TZ 0 cm), a median of 0 GCs/mm (min.: 0; max.: 5.2) was observed. At TZ 5 cm, a median of 21.8 GCs/mm (min.: 4.3; max.: 39.8) was counted. At TZ 10 cm, a median of 26.6 GCs/mm (min.: 7.0; max.: 54.4) was observed. Finally, at TZ 15 cm, a median of 32.1 GCs/mm (min.: 18.5; max.: 45.7) was counted. There was a significant increase in the the number of GCs/mm between 0 cm and 5 cm (*p* < 0.01) and between 0 cm and 10 cm (*p* < 0.001) **(**Fig. 4d**)**. Regarding the differences between the other samples (TZ 5 cm to TZ 10 cm and TZ 10 cm to TZ 15 cm), there was a statistically nonsignificant trend of increasing GC counts, with the difference being the smallest between TZ 10 cm and TZ 15 cm (however, only *n* = 2 for TZ = 15 cm).

## Discussion

In our cohort of 14 HD patients, we assessed the extent of cholinergic innervation in the lamina propria mucosae by AChE scoring, and the number of GCs in colonic biopsies over a maximal length of 15 cm (extending from the proximal area to the beginning of the macroscopically defined transition zone).

The amount of GCs was in a similar range to those amounts reported by Meier-Ruge and Brunner [17]. By using LDH stainings, 7–11 GCs/mm in dys−/or hypoganglionic colon segments, as well as 14.5 GCs/mm in unaffected colon tissues, were reported [17]. Our slightly higher cell numbers may be related by the different staining protocol (NADH instead of LDH). Depending on the staining method as well as the inter-observer variability, GC counts can vary considerably [14, 21]. Swaminathan et al. summarizes the variation of cell counts depending on the staining method ranging from 5 to 756 GCs/cm [21]. Even with laborious counting of the entire circumference, patient-to-patient variation remained very high [22], suggesting that either significant variation in myenteric GC density is normal or variation in technical factors (e.g., stretch) might be relevant [21]. The question whether the dilatation of the transition zone and the oral part of healthy gut leads to neuronal death or whether a reduced cell count is the result of dilatation and stretching (the same amount of CGs distributed in a wider circumference) remains unsolved. During TAPT surgery, longitudinal stretching seems to be more prominent than intravital circumferential stretching, which is difficult to assess. Another factor is tissue shrinking during the embedding process, which is well known from FFPE tissue. This can be neglected in cryoconserved material as in our case. Current literature reflects on the disrupted craniocaudal migratory behaviour of the GCs – and a locoregional partial phenotype which leads to the reduced amount of GCs in the transition zone rather than technical factors [23]. Additionally, the work by White and Langer nicely corroborates the findings on circumferential GC count differences [22]. Swaminathan and Kapur examined this issue in non-HD cadaveric specimens and found that almost the entire circumference of 5 transverse sections from a single sample had to be counted to get an accurate estimate of GC density [21]. Nevertheless, all published staining and counting techniques were reported to have high sensitivity and specificity in an experienced laboratory with accuracy rates as high as 99% [14]. Conventional staining techniques do however not discriminate between GCs and glial bystander cells in the ganglia, which may result in further variation that is caused by inadequate GC identification.

Our surgical specimens were sampled in a longitudinal orientation (from aboral to oral) and the beginning of the TZ was macroscopically defined based on the end of the constricted gut. Two phenomena may affect GC counts: (a) concerning the circumference, some authors have observed a varying, uneven distribution of the GCs in the gut wall (see discussion above) [21] and (b) the beginning of the TZ (“0 cm TZ biopsies”). “0 cm TZ” in our study was macroscopically defined thereby still mainly showing aganglionic bowel segments. In contrast, defining the beginning of the transition zone based on microscopic criteria, a “5 cm TZ biopsy” might be randomly located at 0–5 cm from the aganglionic segment boundary. To reduce the impact of these uncertainties, further research studies should consider an evaluation of intervals that are shorter than 5 cm. As expected from normal embryonic development, our samples showed a longitudinal increase of GCs in oral direction. We found significant changes in the transition zone between TZ 0 cm and TZ 5 cm, followed by a trend of increasing GCs in more proximal zones. Kapur analyzed the relationship between the length of the partially aganglionic transition zone, compared to the length of the aganglionic segment, in 59 patients and stated that, excluding the very long aganglionic segments, the hypoganglionic transition zone was consistently less than 5 cm in length [24]. Nevertheless, there were significant methodological differences in the study design: the criteria used for the diagnosis of myenteric hypoganglionosis on H&E-stained paraffin sections were descriptive and designed to detected only moderate-to-severe hypoganglionosis [21]. Despite these formal differences and limitations regarding to low number of specimens in our study, our results suggest a relatively longer transition zone with still slightly increasing GC counts in areas TZ 10 and TZ 15.

In our study, high AChE score values (score 2/3) were only observed in aganglionic bowel. Absent or low AChE score values (score 0/1) were mostly associated with a high number of GCs, but the absence of cholinergic innervation did not prove a normal innervation. Mild cholinergic innervation has been described in rectal biopsies from patients who did not suffer from HD, possibly because mild cholinergic innervation is part of the normal variation. Little is known about mild persistent cholinergic innervation in the LP in HD patients. It is possible that rare AChE-positive neurites also resolve with the maturation of the child or persist as a normal variant in older children or adults. Finding rare AChE-positive neurites protruding into the LP is described to be a relatively common finding in suction biopsies from pediatric patients who do not suffer from Hirschsprung disease [25–28]. Our findings question that a low amount of remaining AChE positive fibers (AChE score 1) has to be considered as a reliable pathological feature in HD patients. No significant correlation was observed between ganglion cell density and absent (AChE score 0) versus AChE score 1, which extended up to 15 cm proximally to the aganglionic segment. In four cases, we did not identify an area with an AChE score of 0. In these cases, some degree of cholinergic innervation remained, despite a resection margin of up to 15 cm proximal from the TZ. The lack of correlation between ganglion cell density and low AChE score may be based on the variables discussed above: variability in ganglion cell counts, different staining protocols, inter-observer variability, tissue stretching and others, or, alternatively, because AChE score 1 might be part of the normal variation. In our study, we exclusively evaluated transmural biopsies for both initial diagnosis as well as during definite surgery, while in other centers, mucosal or submucosal biopsies may be performed, thus resulting in the risk of insufficient tissue being collected for definite diagnostics [15]. Because no definite abnormal cholinergic innervation pattern (AChE score 2/3) was found in the hypoganglionic bowel of most HD patients, mucosal AChE activity alone should not be used to define the proximal transition zone.

Nevertheless, the definition of the end of the transition zone and the beginning of the normal colon by the use of GC count cut-off values may not lead to improved surgical patient outcomes, with respect to the inter-observer variability and methodological differences. Based on our results, both mucosal and submucosal tissues should be evaluated, however the choice of the resection margins requires further interdisciplinary discussions.

## Conclusion

In our study, we examined the relationship between the amount of cholinergic mucosal innervation and the density of myenteric ganglion cells in resected intestinal specimens from HD patients. Although we identified an association between absent GC counts (aganglionosis) and moderate or high AChE activity, no statistically significant correlation between AChE activity and ganglion cell counts in proximal, ganglionic intestinal segments could be established. Thus, the presence of sparse cholinergic nerves in the mucosa is not a reliable marker of myenteric hypoganglionosis and transmural biopsies are essential to quantify ganglion cells to exclude non-AChE-related features of transition zone. Our data support the high validity of transmural colon biopsies for HD diagnostics especially for cases lacking one of the classic features and highlights the necessity of a close collaboration between pediatric surgeons and (neuro)pathologists.

## Supplementary information

**Additional file 1: Table S1**. GC count and length of corresponding MP segment according to AChE score (Swiss Role specimen). **Table S2**. AChE score, GC count and length of the corresponding MP segment in the transition zone

## Data Availability

The dataset supporting the conclusions of this article is included within the article (and its additional files).

## Abbreviations

AChE: Acetylcholinesterase
f: Female
GC: Ganglion cell
H&E: Hematoxylin and eosin
HD: Hirschsprung’s disease
LDH: Lactate dehydrogenase
LP: Lamina propria mucosae
m: Male
MP: Myenteric plexus
NADH: NADH tetrazolium reductase reactions
TZ: Transition zone
TAPT: Transanal pull through

## References

[1] Borrego S, Ruiz-Ferrer M, Fernandez RM, Antinolo G (2013). Hirschsprung's disease as a model of complex genetic etiology. Histol Histopathol.

[2] Best KE, Addor MC, Arriola L, Balku E, Barisic I, Bianchi F (2014). Hirschsprung's disease prevalence in Europe: a register based study. Birth Defects Res A Clin Mol Teratol.

[3] Friedmacher F, Puri P (2013). Classification and diagnostic criteria of variants of Hirschsprung's disease. Pediatr Surg Int.

[4] Gfroerer S, Rolle U (2015). Pediatric intestinal motility disorders. World J Gastroenterol.

[5] Das K, Mohanty S (2017). Hirschsprung disease - current diagnosis and management. Indian J Pediatr.

[6] Collins L, Collis B, Trajanovska M, Khanal R, Hutson JM, Teague WJ (2017). Quality of life outcomes in children with Hirschsprung disease. J Pediatr Surg.

[7] Brooks AS, Oostra BA, Hofstra RM (2005). Studying the genetics of Hirschsprung's disease: unraveling an oligogenic disorder. Clin Genet.

[8] de Lorijn F, Boeckxstaens GE, Benninga MA (2007). Symptomatology, pathophysiology, diagnostic work-up, and treatment of Hirschsprung disease in infancy and childhood. Curr Gastroenterol Rep.

[9] Edery P, Lyonnet S, Mulligan LM, Pelet A, Dow E, Abel L (1994). Mutations of the RET proto-oncogene in Hirschsprung's disease. Nature..

[10] Meier-Ruge WA, Bronnimann PB, Gambazzi F, Schmid PC, Schmidt CP, Stoss F (1995). Histopathological criteria for intestinal neuronal dysplasia of the submucosal plexus (type B). Virchows Arch.

[11] Meier-Ruge W (1985). Ultrashort segment Hirschsprung disease. An objective picture of the disease substantiated by biopsy. Z Kinderchir.

[12] Holschneider AM, Puri P (2007). Hirschsprung's disease and allied disorders.

[13] Friedmacher F, Puri P (2015). Rectal suction biopsy for the diagnosis of Hirschsprung's disease: a systematic review of diagnostic accuracy and complications. Pediatr Surg Int.

[14] Schappi MG, Staiano A, Milla PJ, Smith VV, Dias JA, Heuschkel R (2013). A practical guide for the diagnosis of primary enteric nervous system disorders. J Pediatr Gastroenterol Nutr.

[15] Meier-Ruge W, Lutterbeck PM, Herzog B, Morger R, Moser R, Scharli A (1972). Acetylcholinesterase activity in suction biopsies of the rectum in the diagnosis of Hirschsprung's disease. J Pediatr Surg.

[16] Meier-Ruge WA, Bruder E (2005). Pathology of chronic constipation in pediatric and adult coloproctology. Pathobiology..

[17] Meier-Ruge WA, Brunner LA (2001). Morphometric assessment of Hirschsprung's disease: associated hypoganglionosis of the colonic myenteric plexus. Pediatr Dev Pathol.

[18] Knowles CH, Veress B, Kapur RP, Wedel T, Farrugia G, Vanderwinden JM (2011). Quantitation of cellular components of the enteric nervous system in the normal human gastrointestinal tract--report on behalf of the gastro 2009 international working group. Neurogastroenterol Motil.

[19] Knowles CH, De Giorgio R, Kapur RP, Bruder E, Farrugia G, Geboes K (2009). Gastrointestinal neuromuscular pathology: guidelines for histological techniques and reporting on behalf of the gastro 2009 international working group. Acta Neuropathol.

[20] Meier-Ruge WA, Brunner LA, Engert J, Heminghaus M, Holschneider AM, Jordan P (1999). A correlative morphometric and clinical investigation of hypoganglionosis of the colon in children. Eur J Pediatr Surg.

[21] Swaminathan M, Kapur RP (2010). Counting myenteric ganglion cells in histologic sections: an empirical approach. Hum Pathol.

[22] White FV, Langer JC (2000). Circumferential distribution of ganglion cells in the transition zone of children with Hirschsprung disease. Pediatr Dev Pathol.

[23] McKeown SJ, Stamp L, Hao MM, Young HM (2013). Hirschsprung disease: a developmental disorder of the enteric nervous system. Wiley Interdiscip Rev Dev Biol.

[24] Kapur RP (2016). Histology of the transition zone in Hirschsprung disease. Am J Surg Pathol.

[25] Schofield DE, Devine W, Yunis EJ (1990). Acetylcholinesterase-stained suction rectal biopsies in the diagnosis of Hirschsprung's disease. J Pediatr Gastroenterol Nutr.

[26] Moore SW, Johnson G (2005). Acetylcholinesterase in Hirschsprung's disease. Pediatr Surg Int.

[27] Pacheco MC, Bove KE (2008). Variability of acetylcholinesterase hyperinnervation patterns in distal rectal suction biopsy specimens in Hirschsprung disease. Pediatr Dev Pathol.

[28] Chow CW, Chan WC, Yue PC (1977). Histochemical criteria for the diagnosis of Hirschsprung's disease in rectal suction biopsies by acetylcholinesterase activity. J Pediatr Surg.

