# Supplementary material for: Cholinergic innervation and ganglion cell distribution in Hirschsprung’s disease
Source: BMC Pediatr. 2020 Aug 24;20:399. doi: 10.1186/s12887-020-02299-z (PMC7445925; doi:10.1186/s12887-020-02299-z)
Supplement: Supplementary file 1 — Additional file 1: Table S1. GC count and length of corresponding MP segment according to AChE score (Swiss Role specimen). Table S2. AChE score, GC count and length of the corresponding MP segment in the transition zone [file 12887_2020_2299_MOESM1_ESM.docx]

| **ID** | **AChE score 0** | | | **AChE score 1** | | | **AChE score 2** | | | **AChE score 3** | | |
| --- | --- | --- | --- | --- | --- | --- | --- | --- | --- | --- | --- | --- |
|  | **GCs** | **length (µm)** | **GCs/mm** | **GCs** | **length (µm)** | **GCs/mm** | **GCs** | **length (µm)** | **GCs/mm** | **GCs** | **length (µm)** | **GCs/mm** |
| **1** |  |  |  | 40 | 3418 | 11.70 | 3 | 3255 | 0.92 | 0 | 2971 | 0 |
| **2** |  |  |  | 97 | 2054 | 47.22 | 4 | 2804 | 1.43 | 0 | 3512 | 0 |
| **3** | 50 | 3069 | 16.29 | 0 | 3095 | 0 | 0 | 3395 | 0 | 0 | 3266 | 0 |
| **4** | 77 | 4290 | 17.95 | 34 | 3353 | 10.14 | 7 | 3477 | 2.01 | 0 | 4460 | 0 |
| **5** |  |  |  | 93 | 4174 | 22.28 | 0 | 3840 | 0 | 0 | 4058 | 0 |
| **6** | 4 | 3680 | 0.92 | 65 | 3543 | 18.35 | 0 | 2708 | 0 | 0 | 4140 | 0 |
| **7** | 63 | 2689 | 23.43 | 4 | 2850 | 1.40 | 0 | 2410 | 0 | 0 | 3150 | 0 |
| **8** | 108 | 3054 | 35.36 | 0 | 3140 | 0 | 0 | 3014 | 0 | 2 | 2497 | 0.80 |
| **9** | 53 | 2724 | 19.46 | 20 | 1017 | 19.67 | 0 | 1744 | 0 | 0 | 3652 | 0 |
| **10** | 49 | 3372 | 14.53 | 0 | 2178 | 0 | 0 | 3150 | 0 | 0 | 2272 | 0 |
| **11** | 25 | 3041 | 8.22 | 0 | 4039 | 0 | 0 | 3204 | 0 | 0 | 2420 | 0 |
| **12** | 68 | 3872 | 17.56 | 0 | 3453 | 0 | 0 | 2149 | 0 | 0 | 3300 | 0 |
| **13** |  |  |  | 78 | 3401 | 22.93 | 0 | 3705 | 0 | 0 | 4450 | 0 |
| **14** | 91 | 4331 | 21.01 | 0 | 2134 | 0 | 0 | 1809 | 0 | 0 | 2434 | 0 |

**Supplementary Table 1: GC count and length of the corresponding MP segment according to AChE score (Swiss Role specimen)**

Abbreviations: AChE score: Acetylcholine Esterase score, GC: ganglion cell, MP: myenteric plexus

| **ID** | **TZ 0 cm** | | | | **TZ 5 cm** | | | | **TZ 10 cm** | | | | **TZ 15 cm** | | | |
| --- | --- | --- | --- | --- | --- | --- | --- | --- | --- | --- | --- | --- | --- | --- | --- | --- |
|  | **AChE-score** | **GCs** | **length (µm)** | **GCs/mm** | **AChE-score** | **GCs** | **length (µm)** | **GCs/mm** | **AChE-score** | **GCs** | **length (µm)** | **GCs/mm** | **AChE-score** | **GCs** | **length (µm)** | **GCs/mm** |
| **1** | 2 | 0 | 3184 | 0 | 1 | 180 | 4519 | 39.83 | 1 | 235 | 4317 | 54.44 |  |  |  |  |
| **2** | 2 | 0 | 3569 | 0 | 1 | 94 | 3752 | 25.05 | 1 | 113 | 2261 | 49.98 |  |  |  |  |
| **3** | 2 | 0 | 3073 | 0 | 1 | 43 | 3794 | 11.33 | 1 | 162 | 3988 | 40.62 |  |  |  |  |
| **4** | 2 | 0 | 3299 | 0 | 1 | 84 | 3981 | 21.10 | 1 | 96 | 4146 | 23.15 |  |  |  |  |
| **5** | 1 | 0 | 3957 | 0 | 1 | 17 | 3919 | 4.34 |  |  |  |  |  |  |  |  |
| **6** | 1 | 56 | 10670 | 5.25 | 1 | 70 | 4507 | 15.53 | 2 | 46 | 6607 | 6.96 | 0 | 82 | 4432 | 18.50 |
| **7** | 1 | 0 | 11000 | 0 | 1 | 169 | 7527 | 22.45 | 2 | 135 | 6853 | 19.70 | 1 | 140 | 3061 | 45.74 |
| **8** | 2 | 0 | 3207 | 0 | 0 | 80 | 2567 | 31.16 |  |  |  |  |  |  |  |  |
| **9** |  |  |  |  | 1 | 174 | 7245 | 24.02 | 2 | 192 | 7628 | 25.17 |  |  |  |  |
| **10** |  |  |  |  |  |  |  |  |  |  |  |  |  |  |  |  |
| **11** |  |  |  |  |  |  |  |  |  |  |  |  |  |  |  |  |
| **12** |  |  |  |  |  |  |  |  |  |  |  |  |  |  |  |  |
| **13** |  |  |  |  |  |  |  |  |  |  |  |  |  |  |  |  |
| **14** | 0 | 0 | 4940 | 0 | 0 | 78 | 4970 | 15.69 | 2 | 170 | 6070 | 28.01 |  |  |  |  |

**Supplementary Table 2: AChE score, GC count and length of the corresponding MP segment in the transition zone**

Abbreviations: AChE score: Acetylcholine Esterase score, GC: ganglion cell, TZ: transition zone, MP: myenteric plexus
